# Applying Proteomics and Integrative “Omics” Strategies to Decipher the Chronic Kidney Disease-Related Atherosclerosis

**DOI:** 10.3390/ijms22147492

**Published:** 2021-07-13

**Authors:** Joanna Tracz, Magdalena Luczak

**Affiliations:** European Centre for Bioinformatics and Genomics, Institute of Bioorganic Chemistry, Polish Academy of Sciences, 61-704 Poznan, Poland; joanna.a.tracz@gmail.com

**Keywords:** chronic kidney disease, cardiovascular disease, atherosclerosis, multiomics, proteomics

## Abstract

Patients with chronic kidney disease (CKD) are at increased risk of atherosclerosis and premature mortality, mainly due to cardiovascular events. However, well-known risk factors, which promote “classical” atherosclerosis are alone insufficient to explain the high prevalence of atherosclerosis-related to CKD (CKD-A). The complexity of the molecular mechanisms underlying the acceleration of CKD-A is still to be defied. To obtain a holistic picture of these changes, comprehensive proteomic approaches have been developed including global protein profiling followed by functional bioinformatics analyses of dysregulated pathways. Furthermore, proteomics surveys in combination with other “omics” techniques, i.e., transcriptomics and metabolomics as well as physiological assays provide a solid ground for interpretation of observed phenomena in the context of disease pathology. This review discusses the comprehensive application of various “omics” approaches, with emphasis on proteomics, to tackle the molecular mechanisms underlying CKD-A progression. We summarize here the recent findings derived from global proteomic approaches and underline the potential of utilizing integrative systems biology, to gain a deeper insight into the pathogenesis of CKD-A and other disorders.

## 1. Introduction

Chronic kidney disease (CKD) is defined as a loss of renal function manifested as a progressive reduction of the glomerular filtration rate (GFR) present for at least 3 months and/or albuminuria, and abnormal kidney morphology [1,2]. CKD is divided into five stages with increasing severity and the transition to stage 3b is the point at which the diseases become irreversible. Patients with stage 5 (CKD5) develop end-stage renal disease (ESRD), display kidney failure with eGFR values below 15 mL/min/1.73 m^2^, and ultimately require a dialysis or kidney transplantation [2,3]. The number of people undergoing renal replacement therapy exceeds 2.5 million and is estimated to be doubled by 2030 [4]. Annual mortality due to CKD increased by 33.7% over the 2007–2017 period [5], and reached similar numbers as colon cancer [6], thereby posing CKD as a one of the main causes of mortality and morbidity worldwide [4,7]. The mortality in CKD, especially in ESRD, is however, not driven by a kidney failure but by the cardiovascular complications caused by accelerated atherosclerosis [8]. CKD patients are exposed to an increased risk of cardiovascular disease (CVD), and its prevalence along with cardiovascular mortality increases alongside declining kidney function [9,10]. Multiple epidemiological studies demonstrated that CKD patients are more prone to develop CVD, triggering over 60% of deaths due to cardiovascular events: stroke or myocardial infarction, while in individuals with normal kidney function this value accounted for less than 30% [11,12]. For ESRD patients, the risk of CVD is 20–1000-fold higher as compared to general population, and raises in proportion to the decrease of eGFR [13]. Nevertheless, even the early stage CKD patients indicate the initial symptoms of CVD, including hypertension or ischemic heart disease and reveal 1.5-fold higher risk of CVD-induced death as compared to general population [9,14]. Cardiovascular complications in CKD are mainly caused by atherosclerosis, and its high prevalence indicates the close functional association of CVD with kidney disease. This phenomenon can be explained by the clustering of several traditional risk factors, including dyslipidemia, hypertension and diabetes, which are characteristic for “classical” atherosclerosis, and the other ones, uremia-related, which are more specific to CKD. In “classical” atherosclerosis, a direct correlation with progress of dyslipidemia is observed. This disturbance is recognized as a fundamental step for the development of complex interplay of hemodynamics, lipid metabolism, and thrombotic processes that initiate chronic inflammation in the arterial wall, and progress towards development of atherosclerotic plaques [15]. In chronic kidney disease-related atherosclerosis (CKD-A), non-traditional risk factors, including accumulation of uremic toxins, inflammation, oxidative stress, malnutrition, disturbances of the calcium-phosphate metabolism, are also considered [16,17]. The effect of uremic and traditional risk factors can accelerate atherosclerosis in CKD [16,18,19]. However, many individuals with CKD reveal a phenomenon called “reverse epidemiology”, and display decreased serum cholesterol, body mass, and blood pressure that are more strongly related to the higher risk of atherosclerosis [20]. All this points towards the non-obvious relationship between CKD and risk of atherosclerosis. In this respect, CKD-A appears as a complex multifactorial disease with many dysregulated processes and pathways. Moreover, CKD patients are often excluded from the large trials of cardiovascular outcomes, well-established diagnostic modalities, and treatment strategies for atherosclerosis and cardiovascular disease [21]. Therefore, studies presenting direct analyses of both conditions, CKD and “classical” CVD, are extremely rare in the literature. The vast majority of them compared CKD to healthy controls, which is the main limitation in unraveling the differences in mechanisms accelerating atherosclerosis in CKD and “classical” CVD. Several excellent reviews have outlined the general features of CKD [22,23]. A report presenting the current state-of-the-art of risk factors and increased cardiovascular morbidity/mortality in CKD has been recently published [24].

In this review, we focus on the utilization of comprehensive “omics” applications, with emphasis on proteomics, to the analysis of molecular mechanisms of CKD-related atherosclerosis. We aimed to underline the possibilities and large potential value of integrative “omics” studies to decipher the disease-related molecular mechanisms. Furthermore, the research on CKD and “classical” CVD and their functional interrelations will aid in better understanding of these mechanisms.

## 2. Key Concepts of Proteomics in Disease Analysis

Proteins are effectors of biological functions and dysregulation of their abundance results in pathological imbalance of an organism. Proteomics aids in the characterization of proteome dynamics, including abundance, function, interactions and modifications of proteins in various types of biological fluids, cells and tissues over time and disease conditions. During the last decade, a broad spectrum of “omics” studies on CKD and CVD, including proteomic ones, has been conducted to determine the specific indicators of disease development.

One of the most common proteomic approaches in analysis of pathological condition relates to the identification of biomarkers. Reviews discussing various “omics” attempts for discovery of clinically relevant biomarkers have recently been published [25,26,27]. Therefore, only a few issues will be mentioned in this review. Protein biomarkers are molecular indicators of perturbed biological processes, physiological pathways, or pharmacological responses to treatment. The knowledge about their level provides useful information for early detection of subtle pathological disturbances and disease progression, and is considered as an introductory phase to focused personalized medicine studies [28]. However, the putative biomarkers have to pass through rigorous validation phase to be implemented in clinical setting. More specifically, the early phase studies have to determine their accuracy, reliability, interpretability, and feasibility in the diagnostic laboratory, with a relatively minimal cost per sample. To determine if a biomarker is “promising”, it is recommended to report its significant association with the clinical outcome and the assessment of sensitivity and specificity [29]. Moreover, the candidate biomarker has to be appropriately validated in vivo, in animal models first and then in cohorts of patients, in independent clinical studies. Normal and prognostic concentration values should embrace the differences matched by gender and age, thereby assisting in clinical decision making [30].

Various proteomic studies have been performed in CKD and CVD, and many putative biomarkers were proposed to aid the diagnosis of CKD or CVD [31,32,33,34]. For instance, neutrophil gelatinase-associated lipocalin (NGAL) and urinary kidney injury molecule (KIM-1) were suggested for an early CKD detection [35,36]. A review that outlined recent advances in the discovery and validation of biomarkers for CKD prediction has been published by Zhang et al. [37]. Most of the studies, however, are not able to meet the above-mentioned criteria mainly due to insufficient accuracy and the sensitivity, low predictive power, the heterogeneous results as well as the lack of validation. An interesting solution that increases the predictive power of biomarkers is a multi-marker strategy, involving combinatorial panel of proteins representing multiple pathways perturbed in a given disease. For instance, the CKD273 classifier, developed by Good et al. [38], is a panel based on capillary electrophoresis/mass spectrometry method (CE-MS), frequently used in the CKD diagnostics, especially on urine samples (reviewed by [39]). On the other hand, lncRNAs may be useful as potential biomarkers for the early diagnosis and prognosis of many diseases, including patients with CKD. The review summarizing the role of lncRNAs in kidney diseases, and their function as prognostic biomarkers has recently been published [40].

Studies targeting single entities might be useful for preventive and therapeutic approaches, and guidelines for biomarker identification and qualification in clinical proteomics have been presented some time ago [41]. Nevertheless, the value of such biomarkers and translatability of preclinical discoveries to human applications is still under debate, and many challenges and bottlenecks are associated with the transformation of biomarkers into potential clinical assays [42,43]. Moreover, many suggested protein biomarkers require detailed mass spectrometry analysis, which might be preclusive for broad utilization in clinical settings on a large scale. To this day, only a few urinary biomarkers, including KIM-1, clusterin (apolipoprotein J), albumin, β2-microglobulin and cystatin C are considered as valid by Food and Drug Administration (FDA) and European Medicines Agency (EMEA). The most suited biomarkers, associated with CKD progression have not been yet discovered and implemented into medical practice. Therefore, proteomic approaches focused on biomarkers searching are relevant, but have limited practicability.

**Figure 1:**
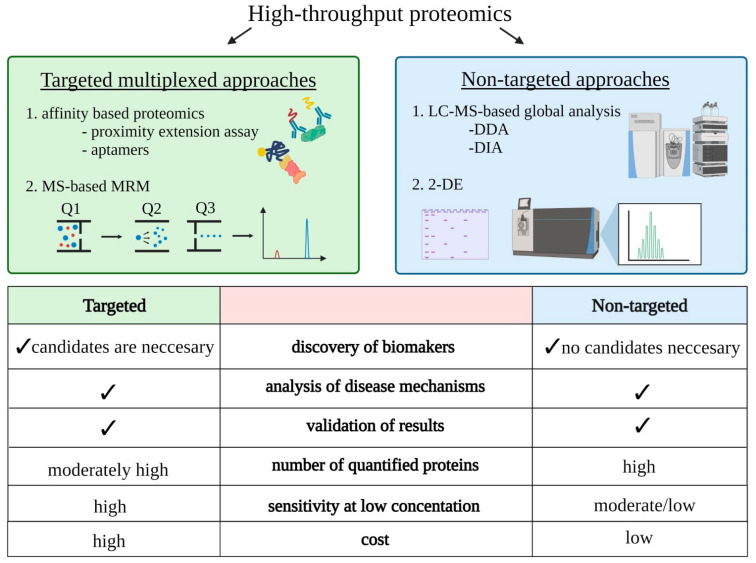
Characteristics of the targeted and non-targeted proteomic approaches. MRM—multiple-reaction monitoring; DDA—data-dependent acquisition; DIA—data-independent acquisition; 2DE—two-dimensional electrophoresis.

However, high-throughput proteomics might be also utilized to recognize complex disease processes and the molecular mechanisms of a pathological condition as well as verification of results obtained with other techniques (Figure 1).

An advancement in proteomic approaches, mainly mass spectrometry techniques and affinity assays, has played a meaningful role in this respect. Comparative proteomic analysis on a large-scale might be useful to create hypotheses, and to determine targets and dynamic interactions between proteins or associations between processes and pathways underlying disease. Such a proteomic approach can utilize many different targeted and non-targeted techniques. Recently, affinity-based proteomics i.e., proximity extension and aptamer-based assays, have been presented as targeted methods to the quantitative, high-specific evaluation of multiple proteins [44,45]. These techniques seem to be very promising especially in analysis of low-abundance proteins, in samples with wide dynamic range of protein concentrations, i.e., plasma or serum.

In proximity extension assay (PEA), a pair of oligonucleotide-labeled antibodies binds to epitopes on the surface of a protein [46]. After the antibodies recognize target molecule, the oligonucleotide sequences hybridize, and the added DNA polymerase leads to an extension of the oligonucleotide sequence. The reaction product is detected and quantified by real-time PCR and the result converted into the amount of examined protein [44]. The immunoassay is multiplexable and can detect up to 92 protein biomarkers in 96-plex format simultaneously without a loss of specificity [47]. Aptamer-based proteomic technology is based on single-stranded oligonucleotides linked with a fluorophore and a photocleavable linker to capture target proteins from a biological sample with a high specificity without utilizing antibodies. The new generation of aptamers, called “SOMAmer” (Slow Off-rate Modified Aptamer), is capable to bind proteins with subnanomolar affinities and without any nonspecific interactions. SOMAmers can be combined in multiplexed detection assay (called SOMAscan), and in this form efficiently quantify more than a thousand proteins at a very low limit of detection, high reproducibility and utilizing a minimal amount of biological samples [45].

Mass spectrometry-based proteomics represents one of the most sensitive methods utilizing to untangle the molecular mechanisms of a disease. This technique can be applied in targeted and non-targeted mode. Application of targeted multiple-reaction monitoring (MRM) mass spectrometry allows specific, highly reproducible and sensitive quantitative analyses of hundreds of proteins in a single experiment [48,49]. It offers not only relative but also absolute quantitation of proteins when isotopically labeled internal standards are utilized. Importantly, the identification of low-abundance proteins in samples with wide dynamic range of protein concentrations is possible. However, being a targeted method, only a limited number of known proteins can be evaluated, which can be an obstacle when a rare biological condition is explored. In consequence, MRM approach is mainly utilized for biomarker validation studies of newly identified protein targets.

**Figure 2:**
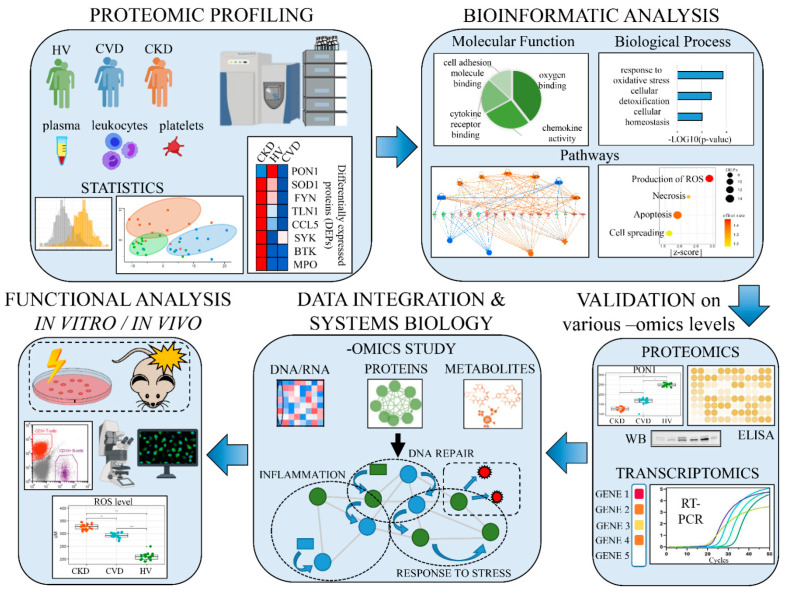
The analysis pipeline targeting the molecular mechanisms of CKD-A utilizing an integrated multiomics strategy.

Non-targeted MS-based methods does not reveal this obstacle and enable the identification and quantitation of thousands of proteins making it an ideal approach in elucidation of the underlying molecular events associated with disease development and progression. However, identification of low-abundance proteins in complex samples is sometimes challenging. The obtained datasets of differentially expressed proteins (DEPs) among multiple biological conditions serve as an input for subsequent functional bioinformatics analyses linking changes in protein abundance with information about disturbed processes and interaction between them in studied pathological condition. Identified DEPs are analyzed in the context of Gene Ontology (GO), and proteins are assigned to particular biological process, molecular function or cellular component categories. KEGG (www.genome.jp/kegg/pathway.html; accessed on 16 March 2021), Reactome (https://reactome.org/; accessed on 16 March 2021), BioCarta (https://maayanlab.cloud/Harmonizome/dataset/Biocarta+Pathways; accessed on 16 March 2021) are most commonly used. In the next step, the enrichment analysis is performed to examine whether any identified functional GO-term in the data set is statistically enriched in relation to its frequency in the genome [50]. Moreover, biological process and molecular function analysis might be fortified with physiological pathway analysis, which is more meaningful since it provides more detailed information about the changes. Identification of the disturbed pathways between two biological conditions supports clarifying the pathophysiology of disease progression. Among the tools for the GO-terms identification and enrichment, open-source software: DAVID (https://david.ncifcrf.gov/), PANTHER (http://pantherdb.org/; accessed on 16 March 2021), and ConsensusPathDB (http://cpdb.molgen.mpg.de/; accessed on 16 March 2021) can be highlighted [51,52,53]. Some commercial software, i.e., the Ingenuity Pathway Analysis (IPA; Ingenuity systems, Redwood City, CA, USA; www.ingenuity.com; accessed on 16 March 2021) and Advaita (www.advaitabio.com/ipathwayguide; accessed on 16 March 2021), have additional built-in scientific literature-based databases allowing indicating the directionality of the observed changes and thus predicting an inhibition or activation of identified pathways/functions. Moreover, these programs allow the functional interpretation of the results derived from other “omics” studies, gathering the information at the gene, transcript and metabolite levels, providing a more comprehensive molecular view of examined biological processes (Figure 2).

Genomics reveals specific mutations related to disease prognosis. Transcriptomic analyses indicate whether the disturbed abundance identified at the protein level is directly related to changes in mRNA expression or arises from post-translational machinery. It is difficult to obtain a good correlation between the level of mRNA expression and the observed abundance of the associated proteins [54]. However, the recent study presented the possibility of predicting protein copy numbers from the transcriptome analysis by considering RNA-to-protein (RTP) conversion factor across several human tissues and cell lines [55]. Metabolomics and lipidomics reflect the metabolic status of cells or tissues but are sensitive to various stimuli, which results in a large degree of variability of observed changes. Nonetheless, combination of all aforementioned approaches in biomedical research delivers one of the most powerful methodologies to understand the complex molecular mechanisms of disease progression (Figure 2). The integration of such approaches constitutes a starting point for functional validation and physiological experiments targeting the unraveled mechanisms, in vitro in cell lines or in vivo, using animal models of studied pathological condition.

In recent years, the number of systematic studies utilizing multi-approach has increased considerably. An excellent example of such a comprehensive pipeline to understand the molecular mechanism of selected aspect of atherosclerotic plaque development is presented in the study by Venturini and co-workers [56]. In this study, the authors have integrated high-throughput proteomics and metabolomics with bioinformatic analysis supplemented with physiological experiments to demonstrate the differences occurring in endothelium under atheroprone and atheroprotective shear stress, a hemodynamic force playing a role in atherosclerosis development. Alterations in lipid and lipoprotein abundance and metabolism at proteome and metabolome levels were presented and the key molecules involved in response to atheroprone flow were identified. The low-density lipoprotein receptor (LDLR) demonstrated a significant downregulation in its abundance and disturbances in the glycosylation pattern, and the changes were validated by immunohistochemistry and Western blot analysis. Moreover, by utilizing an integrative “omics” approach, a modulation of LDLR with statin was shown to be partially recovering the normal, atheroprotective profile of endothelial cells, thereby pinpointing to a new treatment strategy and demonstrating pleiotropic effect of statin activity [56].

## 3. Application of Proteomics to Understanding Molecular Mechanism of CKD-A

Such comprehensive analyses mentioned above, are not very frequent in CKD-related atherosclerosis as researchers focus rather on biomarker searches than on high-throughput and multilevel elucidation of molecular changes. Thus far, only a few studies utilizing the global proteomics and functional bioinformatics, to the study of biological processes and pathways disturbed during CKD and CKD-A progression have been published (Table 1).

### 3.1. Proteomic and Lipidomic Analyses on Lipid Metabolism in CKD-A

Among the major factors contributing to the risk of atherosclerosis are disturbances in lipid metabolism and transport. In the general population, the high concentration of total cholesterol and its atherogenic fraction, LDL, is associated with the pathogenesis of atherosclerosis and the prevalence of cardiovascular events [88]. Additionally, the low concentration of anti-atherogenic high-density lipoprotein, HDL, is regarded as contributing to the CVD progression [89]. Hypercholesterolemia can be controlled by statins, but this treatment is only partially effective in patients with mild to moderate CKD and does not reduce cardiovascular morbidity and mortality at the advanced stage of CKD [90,91,92]. Therefore, nature and molecular mechanism(s) behind dyslipidemia seem to be different in CKD, and this phenomenon is called “reverse epidemiology” [20,93]. In fact, the CKD patients are often characterized by hypertriglyceridemia, which is consistent with symptoms observed in “classical” CVD, however, low total cholesterol, HDL and normal or even low LDL concentration are often observed [94]. Unique dyslipidemia distinguishing patients with atherosclerosis-related and nonrelated to CKD has recently been presented utilizing a shotgun lipidomics approach [57]. The putative functional link between the low cholesterol level correlated with kidney dysfunction supports the postulated “reverse epidemiology”. On the other hand, the upregulation of triacylglycerols in CKD and downregulation of cholesterol/cholesteryl esters, sphingomyelins, phosphatidylcholines, phosphatidylethanolamines and ceramides as compared to CVD group underlines the differences between CKD-A and “classical” CVD.

In addition, proteomic approaches have proven to be very useful in elucidation and providing insights into these alterations in CKD. In CVD non-related to kidney dysfunction, HDL particles have strong anti-inflammatory, antioxidant and antithrombotic properties [89,95]. On the other hand, many proteomic studies suggested that HDL may become dysfunctional or even pro-inflammatory in CKD due to chemical modification or remodeling of its protein composition [58,59,60,61,62]. Among others, the changes in the HDL proteome in ESRD patients in comparison to control subjects were identified and linked with the dysregulation of lipoprotein metabolism, immune response, or platelet activation [61,62]. Moreover, altered protein components of HDL were positively correlated with vascular calcification in CKD and this phenomenon was also related to atherosclerosis progression and CKD severity [63,68]. More importantly, similar alterations in HDL protein composition were identified when plasma proteome of CKD patients was compared to non-renal CVD patients, suggesting dysfunctionality of HDL [64]. Combination of global proteomic profiling with bioinformatics and functional studies of monocytes, together with physiological experiments and validation of obtained results, confirmed defective anti-inflammatory activity of HDL in ESRD patients [60]. Therefore, a link between dysfunctional character of HDL, enhanced inflammation and cardiovascular mortality in CKD was indicated. Complementary proteomic approach based on screening and targeted mass spectrometry demonstrated that the protein cargo of HDL was significantly altered in ESRD subjects [61]. The changes in HDL proteome were furthermore accompanied by derangements in HDL lipid composition, mainly by decreased phospholipid and increased triglyceride content as related with functional impairment of HDL in CKD patients [59]. By utilizing the targeted proteomic approach for analysis of HDL fraction in CKD, it was shown that HDL might also lose its atheroprotective functionality evoked by chemical modifications, such as acylation or palmitoylation that negatively affect reverse cholesterol transport, and can be associated with the high cholesterol efflux [66,96,97]. Recent studies have demonstrated that oxidative stress can modify the HDL protein components leading to dysfunctionality of HDL, and thus to non-effective cholesterol efflux and atherosclerosis acceleration [98,99]. Aforementioned changes in HDL composition were not identified in “classical” CVD, but the opposite situation was observed, i.e., the low level of apo A-IV is one of the important factors affecting atherosclerosis development, whereas in CKD the inverse correlation was demonstrated [61,68,100].

Summarizing, comprehensive “omics” approaches supplemented with Gene Ontology analysis and biochemical, and physiological studies revealed that HDL-related dyslipidemia in CKD patients seems not to be related to the HDL level but rather to its chemical nature and thus functional impairment reflected in deficiency of anti-atherogenic and anti-inflammatory properties. As a result, a decline in the cardioprotective features of HDL is observed in CKD, in contrast to “classical” CVD.

Disturbances related to lipids and lipoproteins in kidney disease revealed by comprehensive “omics” approaches were demonstrated not only for HDL plasma particles. The application of multiomics study of murine kidney, combining transcriptomics, high-throughput label-free proteomics, and metabolomics coupled with bioinformatic analysis, confirmed a significant alterations in gene expression levels and protein abundance, primarily of those involved in lipid metabolism [65]. In addition, downregulation of total phosphatidylcholines, phosphatidylethanolamines, and sphingomyelins in age-related CKD mice was revealed, and the vitamin A pathway was implicated in this process.

### 3.2. “Omics” Strategies to Decipher the Vascular Calcification Mechanism in CKD-A

The vascular calcification, as a pathological deposition of calcium and phosphorus derivatives and other mineral substances in the arterial wall, constitutes the other common metabolic derangement that contributes to the development of CKD-related atherosclerosis [101]. Although, calcification is also observed in “classical” CVD [102], this process seems to be more frequent and extensive in CKD patients due to often occurring mineral and bone disorders and also has prognostic implications [103,104]. Nevertheless, its mechanism remains elusive. Proteomic approaches to tackle the relationship between vascular calcification and atherosclerosis progression in CKD have been undertaken [69,70,71,72]. These studies revealed that vascular calcification is strongly connected with accelerated inflammation [70], and associated with the progression of CKD [67,68,70,105]. Both the vascular calcification and inflammation are strongly exacerbated by uremia and circulating in the blood uremic toxins, i.e., indoxyl sulfate (IS) and p-cresyl sulfate (pCS) [106,107,108]. Exposure of cells to serum uremic toxins in CKD results in morphological alterations and vascular calcification linked with CKD-related atherosclerosis [72,109].

The accumulation of uremia-related factors has been demonstrated to evoke vascular dysfunction in animals and humans with CKD, and was associated with the higher mortality and CVD progression in CKD patients [75,110,111,112,113]. The application of the isobaric tag for relative and absolute quantitation (iTRAQ) label-based proteomics to understand the global alterations during IS- and pCS-treatment revealed that coagulation pathways, hyperglycemia and insulin resistance accompany the development of arterial calcification in CKD model [73]. The deeper insight into the mechanism of IS influence on a pro-inflammatory phenotype and thus atherogenesis in CKD has been proposed by Nakano and co-workers [74]. In this elegant example of integrated “omics” approach, the authors combined global proteomics and transcriptomic analysis to confirm that IS induces inflammation in macrophages of CKD patients. The bioinformatic analyses were used to identify the signaling mechanisms responsible for observed activation of macrophages, and as a result the Notch signaling and the ubiquitin-proteasome pathways were identified. Comprehensive validation on the transcriptome and proteome levels, using Western blotting, flow cytometry and microscope analyses was performed to independently confirm the observed findings. To address the mechanism of disturbed signaling and proteasome pathways, the authors utilized in-depth mechanistic analysis in vitro and in vivo, comprising CKD cell lines and mouse model to follow the molecular events underlying the studied processes. Finally, utilizing siRNA-based silencing technique they identified the particular molecules responsible for transport of IS and its induced effect of the inflammatory processes in macrophages. The presented study constitutes the perfect systems biology approach, combining the number of genomic, transcriptomic and proteomic techniques together with bioinformatics for elucidation of molecular mechanism behind IS-induced inflammation, leading to the progression of atherosclerosis in CKD.

Phosphate can be considered as another uremic toxin involved in vascular calcification, and thus arteriosclerosis and an increased risk of cardiovascular mortality [114]. In CKD, this phenomenon is uniquely functionally related to fibroblast growth factor-23 (FGF23) that strongly increases phosphate level and thus evokes toxic effects on the endothelium and cardiovascular system. The positive correlation between elevated serum FGF23 levels and CVD events, and mortality in ESRD patients has been demonstrated [115]. By utilizing comprehensive analyses based on aptamer-based proteomic platform, combined with the high-throughput metabolomics, glycerol-3-phosphate has been revealed as a potential kidney-derived modulator responsible for the FGF23-mediated disturbances in phosphate homeostasis in humans [69]. Moreover, application of genome editing technology, and in vitro and in vivo mechanistic analysis conducted on cell lines and mouse model resulted in elucidation of a sequence of physiological events underlying this mechanism. This finding provided evidence necessary to understand the molecular mechanism of hyperphosphatemia during the kidney injury and its effect on the vascular system. Moreover, it has also suggested a new therapeutic strategy in atherosclerosis related to kidney disease [69]. Therefore, an evidence supporting the effects of uremic toxins on vascular dysfunction and the progression of atherosclerosis is available, although, the mechanism leading to CVD is not yet fully elucidated.

It has been also suggested that in patients with ESRD, vascular calcification is mediated by decreased ability of vascular smooth muscle cells (VSMCs) to inhibit mineralization process due to vesicle-mediated mechanism [116]. Global proteomic approach in combination with bioinformatics and advanced mechanistic analysis in vitro were utilized to investigate the regulation of matrix vesicles biogenesis in VSMC calcification [80]. The authors demonstrated and validated that VSMC-derived exosomes were involved in this process, and indicated disturbances in calcium ions homeostasis, cell adhesion and motion, and regulation of cell death as the key processes responsible for the observed phenotype. They confirmed the in vitro results utilizing the aortic samples from ESRD patients to present exosomes deposited in pre-calcified vessels and suggested a potential mechanism of the VSMC calcification in CKD, due to increased exosomes release at sites of vascular injury. This study elegantly demonstrated a mechanism that links calcification with the development of CKD-related atherosclerosis.

In another interesting study, the authors utilized “omics” approaches, including proteomics, transcriptomics, metabolomics combined with Gene Ontology analyses to evaluate how the monocyte genome responds to the up- and down-regulation of a miR-223 which is a pleiotropic inflammatory regulator of metabolic-related diseases, including CKD [117]. These results indicate that miR-223 alters osteoclastogenesis and macrophage differentiation via the targeting, among others, the NF-kB pathway and implicates in the progression of calcification process.

### 3.3. Proteomics and Metabolomics in Analysis of Inflammation and Endothelial Dysfunction in CKD-A

The dysfunctional impact of uremic toxins on vascular endothelium plays an important role in CKD-A progression. Due to endothelial dysfunction caused in CKD by inflammation or oxidative stress, the disturbances in cell activation, adhesion and migration occur, and in consequence structural integrity of extracellular matrix (ECM) is altered [118,119,120]. As a result, atherosclerosis and CVD accelerate. Translational proteomic studies have been used to demonstrate the changes occurring in endothelial cells under uremic serum exposure [85,86]. The mechanisms evoked by uremic factors on the endothelial dysfunction and thus atherosclerosis were closely linked to enhanced oxidative stress and induction of pro-thrombotic, pro-inflammatory, and pro-proliferative state. Moreover, histone deacetylase (HDAC), as a key modulator of the uremic-induced endothelial phenotype has been identified [86]. In addition, the authors proposed defibrotide as an endothelial protective compound demonstrating the inhibitory effect on HDAC up-regulation related to uremic medium and suggested the possible mechanism of this inhibition [86].

Combination of metabolomic and proteomic approaches fortified with advanced statistics has also added a piece of a puzzle to this story. Nkuipou-Kenfack et al. [87] pinpointed the alterations in the ECM turnover and fibrosis that occur in the advanced stage of CKD and further enriched the landscape of CKD-A molecular mechanisms. Enhanced endothelial adhesion and transmigration in the uremic conditions have also been demonstrated in study of Trojanowicz and co-workers [121]. The authors exposed a link between ECM, adhesion, transmigration and inflammatory communication signaling pathways and the atherosclerosis progression in CKD. Another insight into these mechanisms derived from a proteomics has been given by Glorieux et al. [81]. In this study, 2054 proteins were identified, and 333 of them displayed an altered abundance in ESRD plasma. The selected results were further verified in an independent cohort of patients using ELISA. Functional analyses performed with IPA and Cytoscape software revealed disturbed pathways related to systemic inflammation, acute phase response, complement and coagulation systems, platelet degranulation, calcium ion-dependent exocytosis, production of nitric oxide and reactive oxygen species. All of these pathways were closely associated with atherosclerosis signaling, moreover, severity of these disturbances correlated with CKD progression [81]. Similar results were presented in our previous studies [67,68,82]. We demonstrated that proteins involved in inflammation, blood coagulation, oxidative stress, vascular damage, and calcification process, exhibited greater alterations in patients with advanced CKD, and that these alterations were connected with progression of atherosclerosis.

Relationship of IS and pCS with 181 cardiovascular-related proteins involved in endothelial function, cell adhesion, complement system, phosphate homeostasis and inflammation, has also been recently analyzed by PEA [75]. Both pCS and IS were positively associated with the increased risk of acute coronary syndrome and with FGF23, involved in CVD morbidity and mortality in renal patients.

The colon microbiome plays a pivotal role in uremic toxins formation in CKD, including IS and pCS [122]. A deeper understanding of this relationship has been recently demonstrated by comprehensive “omics” analysis in CKD rats [83]. The authors showed an altered composition and reduced diversity of the bacterial population, especially depletion of the anti-inflammatory bacteria, as a major cause of inflammation imbalance. These findings were related to changes in blood pressure and kidney parameters, and accompanied by marked alteration of plasma metabolites linked to the inflammation, oxidative stress and metabolism of lipids, amino acids and polyamines. Importantly, possible therapeutic strategy was proposed as treatment with poricoic acid A and fungus *Poria cocos* (*Wolfiporia extensa*) that manipulated microbial dysbiosis and attenuated hypertension, oxidative stress, inflammation, and decline of the renal function in CKD rat model. Finally, some of these results were successfully validated with Western blotting and immunohistochemistry. A relationship between uremic toxins, inflammation, CKD and CVD has been suggested by another, comprehensive “omics” study [76]. In this multi-proteomics approach the global changes in mouse kidney proteome and phosphoproteome upon septic infection were analyzed. Among others, the authors demonstrated that pathways associated with oxidative stress and pyroptosis regulate observed kidney dysfunction. A detailed characterization of the role of inflammation and mechanistic insights into sepsis-associated kidney disease were revealed.

Another interesting study has recently been presented by Yang et al. [77] utilizing modified aptamer based-technology. The authors with IPA software recognized cardiovascular and renal disease- related pathways for the 217 proteins correlated with eGFR, including angiogenesis, control of blood pressure and cardiac fibrosis. Thus, an introduction to the biological basis of cardiovascular relationships with renal and non-renal conditions has been demonstrated. However, taking into account that 1130 individual proteins were measured in this excellent study, in 938 plasma samples, many of these data were unexploited. A few protein predictors of cardiovascular mortality in ESRD patients has been demonstrated recently in the study based on PEA [78]. Nevertheless, majority of data that could have been explored by in-depth bioinformatics were completely omitted.

Specific attention should also be given to diabetes, as a factor strongly associated with the inflammation and development of both CKD and CVD [123]. Although the kidney function decline is one of the most frequent complications in diabetes, nephropathy itself leads also to cardiovascular disease. The relationship between diabetes and CKD has been extensively reviewed, e.g., [124,125]. The pathological link between diabetes and atherosclerosis is also well-documented by others [126]. While the large number of proteomic or even multiomics investigations of diabetic kidney disease (DKD) have been presented, our knowledge about these mechanisms is limited [127,128,129]. This is mainly because diabetes is linked also with a variety of other comorbidities. Thus, studies on atherosclerosis in DKD pose a challenge since it is difficult to separate the effects and reasons behind CKD and CVD, and other atherogenic factors in diabetic patients shall be taken into account. Therefore, the analysis non-diabetic CKD patients seems to be more appropriate, to understand the mechanisms accompanying the progression of atherosclerosis.

### 3.4. The Potential and Future Perspectives of “Omics” Approaches in Understanding Molecular Mechanism of CKD-A

Proteomic analyses of CKD-A offer considerable opportunity in terms of type of samples. A lot of CKD and CKD-A proteomic studies focus on plasma [81,130], and serum [131], although urine comprises the most commonly used type of sample [132,133]. Proteomics of kidney tissue is doable in animal models [134], but in humans such analysis requires taking a tissue biopsy that is difficult to obtain due to clinical or ethical reasons. However, analysis of vessel/aorta samples deriving from surgical interventions in CKD and CVD patients might be useful source of information, especially in the context of calcification process [80]. Unfortunately, these studies are mainly limited to CVD not related to kidney dysfunction so far [135,136].

Additionally, the very low number of proteomic studies was conducted utilizing blood cells, especially leukocytes. As mentioned above, CKD and CVD are closely related to alterations in inflammatory processes, and several researchers demonstrated that plasma/serum proteins involved in inflammation exhibited significant alterations in both diseases [137,138]. However, our previous studies indicated an upregulation of systemic inflammation during CKD development and confirmed that this phenomenon is more pronounced in CKD as compared to the “classical” CVD [68]. Therefore, leukocytes shall be considered as a next source of greater importance in proteomic studies on CKD-A. Scholze and co-workers have analyzed monocytes in non-dialyzed and dialyzed CKD patients, but large-scale “omics” studies have not been performed [139]. Recently, we have demonstrated a complementary proteomic approach to assess CKD and “classical” CVD with distinct atherosclerosis progression. A label-free approach based on LC-MS/MS and functional bioinformatic analyses were used to profile CKD and CVD leukocyte proteins. The dysregulation of proteins involved in different phases of leukocytes’ transmigration process and upregulation of apoptosis-related proteins in CKD as compared to CVD, was presented [79]. We suggested that the disturbances in leukocyte extravasation proteins may alter cell integrity and trigger cell death at the advanced stage of CKD. Importantly, the obtained results were validated by MRM, ELISA, Western blotting, and at the mRNA level by ddPCR and flow cytometry and microscopy analyses.

Similarly, platelets might represent a valuable resource for global/targeted “omics” studies. Dysregulation of coagulation cascade and related proteins has been presented multiple times utilizing patients’ plasma material [68,73,81,82], but according to our knowledge only one study has utilized proteomics on CKD platelets [140]. In this work, the authors demonstrated the alterations in cytoskeletal proteins, presence of the oxidative stress and cell interactions in uremic platelets, the processes also linked with atherosclerosis. Therefore, there is a large hidden potential in the “omics” studies of vessel samples, leukocytes and platelets in CKD-A. Thus, an ideal comprehensive “omics” approach to elucidate the mechanisms behind CKD-A should utilize several types of samples in one study. All mentioned types of samples may easily be analyzed with different proteomic approaches, including LC-MS, CE-MS or, already used less frequently: 2-DE and subsequent MS-based analyses.

Comprehensive proteomic approach, based on different sample specimens and analyzing CKD progression, has very recently been presented by Kim et al. [84]. The authors combined searches of potential urine CKD biomarkers with an analysis of mechanisms underlying the progression from CKD to ESRD. They utilized label-free and label-based quantitative proteomic approaches to investigate the urine proteome changes in the material derived from patients with different stage of CKD progression and rat kidney tissues with moderate-to-severe renal failure. Furthermore, human primary glomerular and proximal tubular epithelial cells were analyzed under hypoxia and normoxia. As a result, proteins related to CKD progression were identified and confirmed by Western blotting, qRT-PCR, and immunohistochemistry. More than 2900 DEPs were identified and utilized for the analysis of cellular pathways and protein-protein interactions associated with CKD severity. Unsurprisingly, immune response and inflammatory processes were upregulated, whereas cell adhesion and migration revealed down-regulation in urine of advanced CKD. Interestingly, activation of cell movement, cell survival, inflammatory response and organization of cytoskeleton, and significant inhibition of fatty acid and nucleic acid metabolism, and regulation of mRNA processing were revealed in injured kidney tissue in rat model. These results were fortified with comprehensive elaboration of proteomic changes associated with fibrosis in hypoxia-treated epithelial cells. This multi-sample proteomics study provides an ideal combination of multiomics approach, and in vitro or in vivo analyses targeting the mechanisms and processes involved in disease progression.

The ongoing rapid development of high-throughput proteomic technologies raises our capacity of large-scale studies leading to the identification/quantification of an increasing number of involved proteins. Since the origination of the proteomics concept, a huge volume of proteomics data has been collected, resulting in rapid development of a plethora of various proteomic bioinformatic platforms and computational tools, including machine-learning approaches. Bioinformatic analysis of biological functions and signaling pathways in the context of their mutual relationships is the foundation for elucidating the molecular mechanisms underlying the disease development and progression. Many high-throughput proteomic studies generating a large datasets involving hundreds to thousands of proteins in a single experiment have been demonstrated also in CKD [45,77,78,141,142]. Unfortunately, despite an unlimited potential and possibilities of these studies, the authors mainly focused on individual proteins/biomarkers. In our view, scrutinizing of a measured protein dataset in comprehensive bioinformatics surveys shall rather serve towards the holistic realization of systems biology approaches, combined with more targeted, functional analysis. This strategy might be useful for providing the complete depiction of molecular mechanisms in CKD-A.

There is also a need to improve the transparency in “omics” research, by including the availability of the raw data and protocols that should be accessible for further use by other scientists. However, due to the complexity of hosting and integration of complete “omics” datasets there is currently no public repository for multiomics data and this limitation has recently been reviewed [143]. Such a multi-platform would add a new possibility and boost a potential of multi-dimensional analysis of diseases. Moreover, to learn how genes, proteins and metabolites interact and influence on CKD-A molecular mechanism(s), new software for integration and analysis of multiomics data should be developed and optimized.

## 4. Conclusions

Despite the failure of proteomics attempts to recognize useful clinical biomarkers in CKD, a number of lessons were learned during these studies, including elaboration of standard protocols for sample proper collection, processing and storage [144]. Moreover, there are enormous, unexploited and already existing datasets derived from multiple CKD-related studies that can be adopted in a meta-analysis utilizing functional bioinformatics in the context of processes and pathways altered during CKD-A acceleration. Combining and a post hoc analysis of these data can provide a systematic picture of CKD-A and elucidation of mechanisms involved in the development of atherosclerosis.

Another unexplored area in CKD-A “omics” analysis relates to leukocyte and platelet as fractions of blood cells strongly associated with inflammatory or coagulation processes and oxidative stress, with suggested involvement in CKD-A development.

Ultimately, although, the close connection between kidney dysfunction and atherosclerosis is widely recognized, and differences between “classical” CVD and CKD-A are underlined, almost all demonstrated studies rely on analysis of either CKD or CVD patients. Taking into account that, especially early stage CKD patients, reveal the same symptoms and take similar medications as non-renal CVD patients, in-depth analysis of both entities conducted in one study may provide more meaningful information about unique character of CKD-A. Interpretation of these results will help in better understanding of the complex pathophysiology and provide novel insights into CKD-A complexity.

## Figures and Tables

**Table 1:** Summary of presented in the literature studies applying “omics” approaches to the analysis of mechanisms in CKD-A.

Studied Mechanism/Process in CKD-A	Applied “Omics” Strategy	References
Unique dyslipidemia	Lipidomics	[57]
Alterations in lipid transport- and metabolism-related molecules	MS-based proteomics Transcriptomics Metabolomics	[58,59,60,61,62,63,64,65]
Disturbances in chemical composition, structure and functionality of HDL	MS-based proteomics Lipidomics	[58,59,60,61,62,63,64,66]
Association of vascular calcification with accelerated inflammation and the progression of CKD	MS-based proteomics	[67,68,69,70,71,72]
Relationships between uremic toxins and development of vascular calcification	MS-based proteomics Aptamer-based proteomics Metabolomics	[69,73,74]
The mechanism of inflammation and atherogenesis development in renal and non-renal conditions	MS-based proteomics Transcriptomics Aptamer-based proteomics	[75,76,77,78,79]
The mechanism of VSMC calcification	MS-based proteomics	[80]
Enhanced inflammatory state or inflammation imbalance	MS-based proteomics	[67,68,76,79,81,82,83,84]
Disturbances in ECM turnover, endothelial adhesion and transmigration	MS-based proteomics Metabolomics Transcriptomics	[79,84,85,86,87]
Dysregulation of hemostasis and coagulation process	MS-based proteomics	[67,68,75,79,81,82,86,87]
The mechanism of hypoxia and oxidative stress	MS-based proteomics Transcriptomics	[84]
